# Late gadolinium enhancement cardiovascular magnetic resonance in genotyped hypertrophic cardiomyopathy with normal phenotype

**DOI:** 10.1186/1532-429X-10-58

**Published:** 2008-12-16

**Authors:** Bradford Strijack, Vignendra Ariyarajah, Reeni Soni, Davinder S Jassal, Cheryl R Greenberg, Robert McGregor, Andrew Morris

**Affiliations:** 1 Department of Medicine, St. Boniface General Hospital, University of Manitoba, Winnipeg, Manitoba, Canada; 2 Division of Cardiology, Department of Cardiac Sciences, St. Boniface General Hospital, University of Manitoba, Winnipeg, Manitoba, Canada; 3 Division of Pediatric Cardiology, Department of Pediatric Medicine, St. Boniface Research Centre, University of Manitoba, Winnipeg, Manitoba, Canada; 4 Institute of Cardiovascular Sciences, St. Boniface Research Centre, University of Manitoba, Winnipeg, Manitoba, Canada; 5 Department of Radiology, St. Boniface General Hospital, University of Manitoba, Winnipeg, Manitoba, Canada; 6 Program in Genetics and Metabolism, Children's Hospital-Health Sciences Centre, Winnipeg, Winnipeg, Manitoba, Canada

## Abstract

A 35 year-old asymptomatic Caucasian female with a family history of hypertrophic cardiomyopathy (HCM) was referred for cardiologic evaluation. The electrocardiogram and transthoracic echocardiogram were normal. Cardiovascular magnetic resonance (CMR) was performed for further assessment of myocardial function and presence of myocardial scar. CMR showed normal left ventricular systolic size, measurements and function. However, there was extensive, diffuse late gadolinium enhancement (LGE) throughout the left ventricle. This finding was consistent with extensive myocardial scarring and was highly suggestive of advanced, non-ischemic cardiomyopathy. Genotyping showed a heterozygous mis-sense mutation (275G>A) in the cardiac troponin T (TNNT2) gene, which is causally associated with HCM. There have been no previous reports of such extensive, atypical pattern of myocardial scarring despite an otherwise structurally and functionally normal left ventricle in an asymptomatic individual with HCM. This finding has important implications for phenotype screening in HCM.

## Case presentation

**Figure 1:**
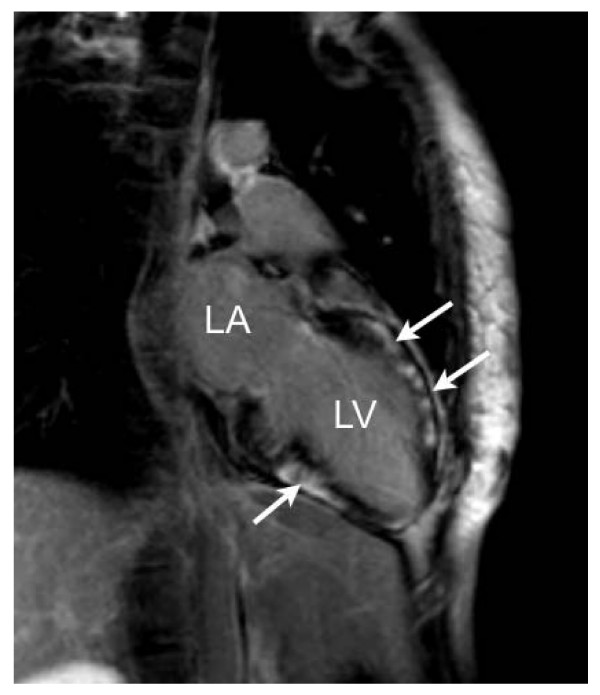
**Inversion-recovery images in the vertical long-axis (2-chamber shown) view demonstrating diffuse, poorly demarcated late-gadolinium enhancement (arrows) without left ventricular enlargement or hypertrophy (LA – left atrium, LV – left ventricle)**.

A 35 year-old asymptomatic Caucasian female with a significant family history of hypertrophic cardiomyopathy (HCM), including sudden cardiac death of unknown cause in one son and HCM in two other children, was referred for cardiologic consultation. The electrocardiogram and transthoracic echocardiogram had revealed no abnormalities. Cardiovascular magnetic resonance (CMR) was performed for further assessment of myocardial function and detection of myocardial scar. Two-, three- and four-chamber CMR cines were obtained using steady-state free precession and inversion-recovery imaging was used to assess late gadolinium enhancement (LGE). CMR showed normal left ventricular (LV) systolic function (end-systolic volume index 24 mL/m^2^; end-diastolic volume index 72 mL/m^2^, ejection fraction 63%) and normal wall thickness (anteroseptal wall thickness 0.8 cm, posterolateral wall thickness 0.8 cm). There was extensive, diffuse, poorly demarcated LGE throughout the LV ventricle, predominantly involving the basal lateral, basal anteroseptal and mid-inferior walls (figures 1A and 1B). Subsequent genotyping showed she was heterozygous for the mis-sense mutation 275G>A (R92Q) in exon 9 of the cardiac troponin T gene (TNNT2) previously reported to be associated with HCM [1,2]. The same gene defect was also found in both surviving children with HCM.

Myocardial scar in HCM shows wide variation. Longitudinal stripes of fibrosis commonly occur at the junctions of the interventricular septum and free wall of the right ventricle (right ventricular insertion points), but more advanced forms include dense replacement scars throughout the ventricle [3]. Myocardial scar in HCM may serve as an arrhythmogenic substrate and the extent of area involved has been shown an association with arrhythmias, sudden cardiac death and major adverse cardiovascular events [3-5]. Diffuse LGE on CMR without left ventricular hypertrophy or enlargement is uncommon in HCM. LGE represents myocardial fibrosis in HCM, and therefore, with such diffuse pattern of myocardial scarring, left ventricular function might be expected to be depressed. The presence of diffuse LGE in a patient with an unequivocal HCM genotype and family history in the setting of an unequivocal normal cardiac phenotype has not been reported previously. This indicates that cardiac fibrosis may be the only demonstrable abnormality in HCM, and precede other phenotypic expression in HCM. This finding has important potential implications for family screening in HCM. Further evaluation of the need to include LGE CMR as routine practice to screen relatives of patients with HCM is required.

## Consent

Written informed consent was obtained from the patient for publication of this case report and accompanying images. A copy of the written consent is available for review by the Editor-in-Chief of this journal.

## Competing interests

The authors declare that they have no competing interests.

## Authors' contributions

BS wrote drafted the manuscript. VA interpreted the cardiovascular magnetic resonance images and was responsible for the idea for the manuscript, performed sequence alignment of the manuscript and, helped write and rewrote the manuscript. RS was the pediatrician for the patient's children and subsequently conducted genetic testing on them. CG conducted genetic counseling and testing on the patient, and interpreted the significance of those results. DS and RM prescribed, performed and interpreted the various sequences during cardiovascular magnetic resonance imaging of the patient. AM was the patient's cardiologist and had been responsible for the patient's care, referral for further testing, manuscript revision and for echocardiographic assessments. All authors read and approved the final manuscript.

**Figure 2:**
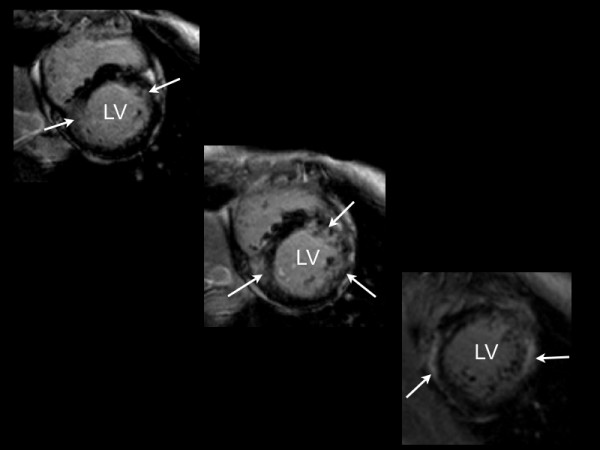
**Inversion-recovery images in the vertical short-axis view demonstrating diffuse, poorly demarcated late-gadolinium enhancement (arrows) without left ventricular enlargement or hypertrophy (LA – left atrium, LV – left ventricle)**.
